# Psycho, Pharmaco and Sex therapy for the treatment of Premature Ejaculation

**DOI:** 10.12669/pjms.38.8.5217

**Published:** 2022

**Authors:** Syeda Razia Bukhari

**Affiliations:** 1 Dr. Syeda Razia Bukhari, Consultant Clinical Psychologist, Higher Education Commission (HEC, Pakistan) Approved PhD Supervisor, Assistant Professor & Student Counselor, Faculty of Education and Social Sciences, Dept. of Social Sciences, Shaheed Zulfikar Ali Bhutto Institute of Science and Technology, H-8/4 Islamabad, Pakistan

**Keywords:** Sex therapy, Combination therapy, Behavior therapy, Psychotherapy, Premature ejaculation (PE)

## Abstract

Pakistani’s men with Premature ejaculation (PE) hardly prefer to go for treatment due to cultural barriers (men dominating society). This research objective is the gives knowledge about the effective treatment of premature ejaculation, how it can be effectively treated with the help of different techniques. Premature ejaculation (PE) is happened when a man has an orgasm and ejaculated earlier at the time of intercourse then he or his spouse would like. PE is a men sexual dysfunction that creates a significant anguish for men, partner and their relationships’. Premature ejaculation is not only one illness; it contains 4 subtypes with a unique psychological concern and problems (longtime, acquired, organic and subjective). PE men and couples are treated psychologically for sexual ability but also for self-esteem, performance anxiety and interpersonal disputes. Psychotherapy outcomes alone are hard to compare and to appreciate due to insufficient methodology (lacking control groups, limited sample sizes, poor results and insufficient monitoring). Rare researches, however, which overcome these methodological barriers, indicate that psychological intervention provides a promising treatment choice for men and partners. A detail literature review was done via an electronic database, “Cochrane library databases”, “PsychInfo”, and “PubMed” from June to July 2021. The most effective procedure for lifetime and acquired PE is pharmaco- and psychotherapy combination. Men and partners develop sexual abilities and resolve the intra-psychological, interpersonal and cognitive problems that trigger and sustain dysfunction.

## INTRODUCTION

Premature ejaculation (PE) is a men’s sexual condition which causes considerable distress to the individual, his partner and his relationship.^1^ Exact etiology of PE cannot be defined as biological (hereditary, somatic, neurobiological) or psychological (interpersonal, cognitive) characteristics.^1^ Many researches indicated high frequency of PE in Pakistan,^2^,^3^ and hardly they preferred to go for treatment due to cultural barriers. This review article is about PE and way of treatment of premature ejaculation and how it can be treated effectively.

### Premature ejaculation (PE) subtypes:

The classification of PE has passed through many variations, beginning with the vague, unreliable, subjective and non-reliable meanings from clinician to other.^4^ The most recent description found in the 5th Edition of Mental Disorders (DSM-5) diagnostic and statistical manual^5^ are stated below:

- PE (302.75).
- Continued or frequent ejaculation patterns in spousal sexual interaction within 1 minute before penetration of sex;
- The symptoms in Criterion A must be present in almost all or nearly all of the cases (about 75-100 percent) of sexual contact (in specified circumstances or generalized, in all context) and must last at least 6 months;
- Criterion A symptoms are causing the person clinically serious distress;
- No sexual dysfunction or due to some substance/medicines or some other medical problem is better characterized for nonsexual psychological disease or because of severe interrelationship depression or other significant stressors.

### Specify:

- ***Severity:*** mild, moderate or severe.
- Lifelong vs. acquired;
- Generalized vs. situational;

The DSM-5 description includes many strengths and limitations. The clear objective criteria are its strongest strength, especially the demonstrated intravaginal ejaculative latency cut-off (IELT). The classification of DSM-IV-preceding TR was vague, unspecific as well as ambiguous.^6^ DSM-IV defined ejaculation as ’on or shortly after penetrating and before the individual wants it.’ Ejaculation is a matter of fact. The accuracy of the DSM-5 criterion would boost the reliability and consistency between clinicians performing ejaculatory disorder research. The DSM-5 term, one of its drawbacks, covers both lifetime and acquired types of PE. IELT population-based research of Men with lifetime PE,^7^ have been performed; however, there is no evidence-based studies to reduce IELT acquired PE. There is still a debate as to whether IELT cutoffs similar or different should refer to PE’s lifetime and inherited subtypes. At what stage the IELT decline from normal to abnormal occurs, the most relevant question for acquired PE. Nevertheless, the PE Guidelines Committee proposed an alternative suggestion that PE acquired would be clinically relevant and often disturbingly reduced by around 3 minutes or less; DSM-5 indicates a similarity.^8^

The truthfulness of the DSM-5 or ISSM (International Society for Sexual Medicine) or an alternate description of the PE obtained will be determined in further study. Another drawback of DSM-5 is the heterosexual prejudice, as it only applies intravaginally and disregards males who are sexually related to men or others. Correlations between masturbation, coital and oral sex are generally not always high.

In contrast to life-long and acquired classes of PE,^9^ Waldinger proposed two other subtypes: VPE (variable premature ejaculation) and SPE (subjective premature ejaculation). Subtypes should be seen as temporary but identify men who do not follow DSM-5 or ISSM, however, who are in trouble seeking help in resolving their ejaculatory problems. VPE (variable premature ejaculation) is recognized by an unusual and inconsistent shorter ejaculatory latency, with the subjective feeling of a reduced ejaculation management. This category is not a sexual disorder but is instead considered to be a natural sexual performance variance. One or more features of the following are SPE (subjective premature ejaculation): (I) an issue of presumed limited ejaculation or lack of control over ejaculation time; (II) subjective perception of stable or incorrect short time IELT; (III) real IELT over the normal range or even further (i.e., a more than five-minute ejaculation); (IV) a reduced or absent capacity to regulate ejaculation (i.e., the ability to postpone ejaculation when ejaculation is imminent). (V) A concern not better described by another mental disorder.^4^,^9^

### Sources and Database:

A detail literature review was done with the help of electronic database, such as “Cochrane library databases”, ”PsychInfo” and “PubMed” from June to July 2021. With the help of electronic database all article were found in English languages which cover the relevant objectives of the current review article. Different keywords have been used for searching such as cognitive behavior therapy for Sexual dysfunction, CBT for Premature Ejaculation, CBT for sexual problem, and Pharmaco therapy for Premature Ejaculation etc.

### General considerations on premature ejaculation psychotherapy:

In today’s PE psychotherapy, psychodynamic, processes, behavioural, and cognitive methods are combined in a short-term psychotherapeutic model.^10^ Ejaculation management, monitor and resolve the increasing impact of PE’s snowball on men, spouses and couples are the fundamental principles of treatment. All common Snowball effects include developing anxiety problems, lower self-esteem, avoiding sexual interaction and partner anger and aggression and a significant decline. Clinicians with various scientific backgrounds have various theories for why an individual suffers from PE. Psychodynamic clinical psychodynamics^11^ have emphasized unconscious aggressive emotional states toward women, pathological narcissism, fears of the vagina that cause them discomfort, passive pleasure of losing urination control and soiling or degrading women. The result of PE is a lack of sensitivity or responses affected by early experiences (e.g., hasty and nervous lovemaking in the car back seat) by behavioural therapists,^12^,^13^ and does not improvise in sexual arousal levels.

Cognitive therapist investigates perception distortions (for example, disaster, overgrowth, mental reading, etc.).^14^ Which retain or aggravate PE. Family and relation counseling specialists focus on the dynamics, power and control issues in each relationship and the management of emotional and sexual intimacy between each of the partners. Many psychotherapists/sex clinicians merge ideas and offer men or partners in different theory schools an interactive format for psychological therapy. Three common factors leading to psychotherapy are recognized by Donahey and Miller.^15^ There are some of them: (I) encouraging the patient to feel that they have the potential to affect and improve their environment; (II) create a positive, empathetic environment where the patient can address psychological and behavioural problems, options and meanings, and (III) optimism and realistic outcome expectations. These three components can be used to treat men and partners with PE psychiatrically.

### Psychotherapeutic treatments for permanent, learned, normal and subjective premature ejaculation (PE):

There are several objectives in the therapeutic treatment of PE. (I) learning techniques to monitor or postpone ejaculation; (II) trust in sexual achievement; (III) lowering anxiety about performance; (IV) amending strict repertoire sexuality; (V) the overcoming of intimacy obstacles; (VI) the resolution of interpersonal dysfunctional problems;(VII) dealing with and interfering with the emotions and thoughts of sexual function;(VIII) Enhanced contact. Sexual psychotherapy is usually known as sexual therapy.^16^ Sex therapists use such therapeutic methods in treating PE, Stop-start or squeeze strategies, for example. Sexual counseling is more than just a combination of therapy; therapists often need to discuss relevant relationship causes, concerns about success and partnership issues. PE psychotherapeutic / sex therapy may be done in pair, person or group formats. The internet or phone^17^ also provides psychoeducational facilities. The Panel proposed different types of treatment procedure^2^ for the long, acquired, intrinsic and subjective styles of PE.

Although the four subtypes clearly overlap in how individuals, partners and pairs view PE subjectively, there are also substantial differences between the subtypes. For example, men with PE in life never had long-term relationships or rarely had normal PE delays. In certain cases, the acquired men are upset more because they realize what is lacking. Lifetime men, but with significant distress for them and their spouses, have accepted their condition. Subjective PE men claim that, amid prolonged sex latencies, they suffer from PE.^17^,^18^

They don’t have the time, they put the sense on their experience. And in the end, natural PE doesn’t understand normal variance in their latencies, and when they undergo rapid ejaculation they have to be overly critical of themselves. These variations among subtypes demand that clinicians tailor their therapeutic interventions with and PE subtype to the general and unique experiences of men and their spouses.

Table-I summarizes the PE recommendations for care provided to all four PE subsystems by the ISSM Guidelines Committee.^2^ Because lifelong PE is focused primarily on the treatment of neurobiology, this is typically a first-line recommendation. Again, the reader can read in this volume the manuals of Dr. Hisasue and McMahon to study PE pharmacotherapy in more detail. In contrast, drug treatment is not recommended if no subjective natural and subjective PE is influenced by biological factors.

**Table-I:** ISSM recommended treatments for the different subtypes of PE.

Subtypes of PE	ISSM recommended treatments
Lifelong PE	Pharmacotherapy, combination psychological & medical therapy
Acquired PE	Treatment of underlying condition, psychotherapy or behavioral therapy, pharmacotherapy alone or, combination psychological & medical therapy
Natural PE	Reassurance, education, psychotherapy or behavioral therapy
Subjective PE	Reassurance, education, psychotherapy or behavioral therapy

PE: premature ejaculation.

Biological conditions such as infection of the prostate or urethra**,** prostatitis and hyperthyroidism can be associated with PE. These circumstances should be dealt with first. If after the treatment of these biological reasons PE persists, both patient and clinician should suggest pharmacotherapy, psychotherapy or combination of drugs and psychotherapy. Reassurance, education and psychotherapy are the proposed methods for treating both natural and subjective PE.^4^,^8^

The pharmacological and mental/constitutional phases of combination therapy include incremental or concurrent application.^18^,^19^ Combination counselling provides men a drug that delays ejaculation when learning therapeutic techniques to delay ejaculation, solve associated psychological and interpersonal issues. After 6 weeks or longer if possible, the drug is weaned because men have proven to have reliable IELT gains. In a combination therapy study men were compared with men receiving dapoxetine 30 mg (a drug approved for treatment of epidemiologic EP in more than 60 countries). Dapoxetine 30 mg 29 was taken in males. The only dapoxetine group showed a two times increase in IELT after 24 weeks, while the combination group increased nearly four times. There are four other reports that indicate that PE 30.33 is superior for men in combined care with medical treatment alone. Despite the reality, clinicians are dependent upon current practices that can be discarded and integrated with different disciplines without formal training. In my view, mixed counseling approaches are used for gender recognition and desire.^10^

### Specific cognitive and behavioural strategies:

People with PE fear they will focus on sexual excitement and think they will ejaculate much more quickly. They use insightful distraction techniques, such as: complex statistics, stock-market investments dreams, sport teams winning/losing records or data from their favorite athletes. Men also drink too much alcohol to lower their sexual enthusiasm. They were probably not be able to obtain or keep an erection as they misconception their alcohol consumption. PE patients often use numerous condoms, desensitizes salts or sprays, or masturbates repeatedly before intercourse. However imaginative, these techniques reduce foreplay enjoyment and generally fail. In combined intimate acts, those men prohibit their partners from touching them and are unhappy about their fear of sexual stimulation. The study of PE male partner distress emphasizes his disrespect for the partner, his overemphasis on performance, short IELT and the absence of ejaculative control.^20^

Because of their anxiety and lack of concentration on their sexual excitement, PE men usually identify that there is no excitement and ejaculation in two points of continuous sexual arousal. Behavioural treatments, including a stop-start, aim to help men reach an excitement level without fear and to stay in the midst of sex. They learn the intermediate phases of sexual excitement by graduated behavioural tasks to understand and familiarize themselves.^21^

Successively, they manage to stay in a medium range of agitation, thus delaying ejaculation, beginning from masturbation and progressing progressively to prevention and sex. Cognitive changes which help sustain the dysfunction are also helpful to fix. The following cognitive distortions are defined in Rosen et al. list^21^: (I) thinking all or nothing, for example, “I’m a complete failure because I’m approaching quickly;” (II) over-generalization, such as “If I had trouble handling my ejaculation last night, I won’t be able to do it today;” (III) the disqualification of the positive, as in “My wife said we’re happy because my feelings aren’t hurt”; (IV) mind reading, as in “I don’t have to ask, I know how you felt last night”; (V) fortune telling, such as ’I know tonight is going to be a disaster’; (VI) emotional reasoning, such as “Everything must be correct because a person believes anything to be true;” (VII) categorical imperatives, such as ‘shoulds,’ ’bought into,’ and ‘must,’ rule the cognitive process; and (VIII) tragic, as in “If I fail tonight, my girlfriend will leave me.^14^,^22^

### Interpersonal issues:

Who was the first to come? The PE caused the interpersonal issues or caused the PE to interpersonal problems. In my experience, the outcomes are both ways. In some situations, the PE has caused interpersonal conflict; in others, the PE has maintained a stable, safe, and loving relationship. PE, on the other hand, is known to wreak havoc on relationships. The men’s PE has dwindled. Some spouses blame their partners (for example, she’s too pretty; I can’t help her), while others are concerned about their partners having extramarital affairs in the future, and still others pursue non-coital means to please their partners only to be rejected.^21^

Female partners are frustrated by the man’s inability or unwillingness to resolve the problem, and they resent his obsession with his foreplay performance. When the man ejaculates, female spouses frequently experience a brief pause in emotional privacy. He looks away, perplexed; she feels betrayed and angry. As previously stated, these issues are continuing to worsen, snowballing into more long-term and contentious issues. Therapists try, while decreasing the aggression and withdrawal, to help the couple recover their emotional and sexual intimacy. The main goals of relationship counseling for PE are to assist partners in overcoming these obstacles, finding practical solutions, and working together constructively to solve problems.^14^

### The effect of Psychotherapy:

The majority of psychotherapy outcome studies deviate from the evidence-based medicine norm. They appear to be unregulated (no waiting list or fake operation), use small samples with insufficient follow-up, and study a variety of PE groups due to definitional disagreements (e.g., in some PE groups the IELT is 2 minutes; in others, it is 6 or more minutes). Few studies in their reviews are included in meta-analytic studies due to a lack of research quality. In one meta-analysis, there was no evidence that physical behavioural interventions improve IELT and other results by waitlist checks;^23^ in two other meta-analyses, psychological PE methods had poor and conflicting data.^23^,^24^ The use of combination therapy is well shown in all three meta-analyses (more efficacious than drug alone).

However, two psychotherapy trials with a limited sample size provide useful information. De Carufel and Trudel^24^ found an eight-fold increase in IELT in the psychologically treated men (Sensuality education, movement of the body, speed of sexual activity, tension and breathing muscles, squeezing and stop/start technique) relative to the wait-list power.^13^ Squeezing, concentrating, individual and joint therapy, sexual education, and communication training^13^ were all documented by Masters and Johnson. Their ’failure rate’ was 2.2 percent immediately after treatment and 2.7 percent after 5 years of follow-up.^13^ There is obviously a need for more research using better sampling, statistics, and results methods.

## DISCUSSION

Psychological procedures generally provide effective treatment options for sexual disorders. In terms of PE, both the rapidity and the stress of the ejaculatory response of the individual, spouse and couple are tackled by psychological treatments. Pharmaco- and psychotherapy combination provides better effectiveness than drug only. Men and couples improve their sexual skills and address the intra-psychic, behavioural, and cognitive issues that lead to dysfunction.^10^,^14^ We’ve improved our outcome metrics even more recently, allowing researchers to better assess the efficacy of their interventions in the field. We are all united as to who does and does not suffer from PE, so that the homogeneity of treatment groups can be improved. Training in sexual skills, partners and cognitive therapy all help to ease the anguish of PE sufferers, men, partners and couples.^9^,^14^,^22^ Hopefully further researches are to be done to help assess the quality of psychological intervention and included in potential meta-analysis.

## CONCLUSION

It is concluded that Premature ejaculation (PE) can be successfully treated with the help of Psychosexual, Pharmaco and combination therapy.
